# Next-generation polydopamine nanocoatings advancing the understanding of surface properties and antimicrobial efficacy

**DOI:** 10.1038/s41598-025-33787-w

**Published:** 2026-01-04

**Authors:** Supriya Nambiar, Dilip G. Nayak, Arun M. Isloor, Ethel Suman, Sooraj Nayak, Shama Prasad Kabekkodu, Rajath U. Rao

**Affiliations:** 1 Department of Orthodontics & Dentofacial Orthopaedics ,Manipal College of Dental Sciences Mangalore, Manipal Academy of Higher Education, Manipal, Karnataka 576104 India; 2 Department of Periodontics ,Manipal College of Dental Sciences Mangalore, Manipal Academy of Higher Education, Manipal, Karnataka 576104 India; 3 Membrane and Separation Technology Laboratory, Department of Chemistry, National Institute of Technology Karnataka, Surathkal, Mangalore, 575 025 India; 4 Department of Microbiology, Kasturba Medical College Mangalore, Manipal Academy of Higher Education, Manipal, Karnataka 576104 India; 5 Department of Cell and Molecular Biology, Manipal School of Life Sciences, Manipal Academy of Higher Education, Manipal, Karnataka 576104 India; 6 Department of Community Medicine, Kasturba Medical College Mangalore, Manipal Academy of Higher Education, Manipal, Karnataka 576104 India

**Keywords:** Polydopamine, Zwitter ion, Surface characterisation, Antimicrobial, Health care, Dentistry, Dental materials, Dental biomaterials

## Abstract

Biofilm formation and bacterial colonization on titanium implants pose significant challenges in healthcare, often leading to implant failure. Surface modifications using nanotechnology offer a promising approach to improve antibacterial properties while maintaining biocompatibility. To evaluate the surface characteristics, cytocompatibility, and antibacterial efficacy of titanium discs coated with polydopamine (PDA) alone versus PDA combined with poly (MBAAm-co-SBMA) zwitterionic nanoparticles. This in vitro comparative study involved the coating of titanium discs into two groups: Group 1 (PDA-coated) and Group 2 (PDA + poly (MBAAm-co-SBMA) zwitterionic nanoparticle-coated). poly (MBAAm-co-SBMA) zwitterionic nanoparticles were synthesized using the distillation–precipitation polymerization method. Surface morphology and Surface Roughness was analyzed using field emission scanning electron microscopy (FESEM) and Atomic force Microscopy (AFM), and elemental composition was determined via energy-dispersive spectroscopy (EDS). Cell viability was assessed using BCA protein assay in, while antibacterial activity against Streptococcus mutans was evaluated using the disk diffusion method. Statistical analysis was conducted using one-way ANOVA with a post-hoc Tukey test (p < 0.05), and results were reported as mean ± standard deviation . FESEM revealed uniform nanoparticle deposition with globular morphology PDA + poly (MBAAm-co-SBMA) zwitter ion nanoparticles. EDS confirmed increased carbon presence in the zwitterion-coated group. Cell viability was comparable between PDA (49.1%) and PDA + poly (MBAAm-co-SBMA) zwitterion (52.5%) groups. PDA + poly (MBAAm-co-SBMA) zwitterion group showed a significantly reduced S. mutans colony count (1.25 × 10⁴ CFU/mL) versus Group 1 (1.4 × 10⁵ CFU/mL). Conclusion Even though Polydopamine has significant antibacterial activity as evidenced in literature, it was observed in this study that, PDA-poly (MBAAm-co-SBMA) zwitterionic nanoparticle coatings demonstrated superior antibacterial activity and favourable surface morphology than PDA, without compromising cytocompatibility, making them suitable for reducing biofilm-associated infections on titanium implants.

## Introduction

The oral cavity, which contains water, saliva, and gingival crevicular fluid, is a very demanding environment for dental materials, implantable or otherwise, since it is in continual contact with them. In hospitals and clinics, increased chances of infections especially from methicillin-resistant staphylococcus bacteria pose a serious threat because of its high infectivity. Medical and dental implantable devices, carry a high risk for these frequent bacterial infections, which can have major negative consequences and raise the patient’s socioeconomic burden. Typically, *Streptococcus* sp., *Staphylococcus* sp., *Escherichia* sp., and *Pseudomonas* sp. are the primary cause of infections in the oral cavity. In addition, hospital and clinic surfaces can be a source of several infections leading to several hospital related infections^1,2^.

Most of these hospital acquired infections are antibiotic-resistant, which is a huge global healthcare burden. This concern about antibiotic resistance has in turn led to a lot of experimental research on antibacterial coatings which can reduce the reliance on antibiotics. Since antibacterial coatings can help in reducing the spread of pathogens, it can be seen as a modality to assuage a major public health concern^3^. There has been several attempts to enhance the surface properties of the biomaterials with various antibacterial coatings, reducing the likelihood of infection. Antibacterial coatings provide us with a more proactive and less invasive approach towards better product life and infection control, thereby leading to better public health. Antibacterial coatings is a major area of research in dentistry, especially because of the microbiome of the oral environment and the need for a lot of implantable devices used in dentistry^4^^[,5^.

Advances in nanotechnology and nanomaterial science is driving the development of effective and better antibacterial coatings^6^. Bacteria exist in two forms: free-floating (planktonic) and as structured communities within biofilms. Biofilms, particularly in the oral cavity, offer bacteria a highly protective environment, making them more resistant to treatment. Following implant placement, a critical competition arises between host cells and bacteria for surface colonization. Favourable host cell adhesion reduces the risk of infection, whereas bacterial dominance can lead to complications. This understanding has driven efforts to modify implant surfaces to enhance biocompatibility, promote tissue integration, and resist biofouling. However, incorporating antibacterial agents often compromises the mechanical integrity of these materials, posing a challenge for their clinical application^7,8^.

Polydopamine (PDA) is a bioinspired polymer that mimics the adhesive proteins of mussels, which can bind to both dry and wet surfaces via catechol and amine groups. It’s simple coating process, high biocompatibility, and ability to adhere to a wide range of substrates make it ideal for surface modification, including metals and biological tissues^9,10^. PDA exhibits free-radical scavenging, antioxidant, antimicrobial, and corrosion-resistant properties, along with strong metal-ion chelation and photothermal conversion efficiency^11^. It can form coordination bonds with metal ions, enhancing the properties of metal alloys. PDA also forms effective composites with nanoparticles, offering potential in biomedical and orthodontic applications such as preventing white spot lesions and improving gingival health through improved surface biocompatibility^8,12,13^.

Zwitterion materials possess both cationic and anionic groups with high dipole moments, yet maintain overall electrical neutrality. This enables them to form a stable hydration shell via strong electrostatic interactions, more robust than hydrogen bonds, tightly binding water molecules^14^. Zwitterionisation of polymeric and metal surfaces has achieved ultra-low fouling levels of < 5 ng/cm², as reported by Colila et al.^15^ and Ding et al.^16^ The term “zwitter” in German means “hybrid” or “hermaphrodite,” reflecting their neutral nature. These ions exhibit both acidic and basic properties and can originate from amphoteric compounds. Zwitterions are categorized based on their ionic arrangement; betaine-like types dominate, combining quaternary ammonium cations with phosphonates, sulfonates, or carboxylates^17^. Medical-grade implants and scaffolds, typically fabricated via rapid prototyping, are highly porous^18^. Zwitterionic biomaterials such as pure nanocrystalline hydroxyapatite and Ti6Al4V 3D-printed scaffolds have shown effective S. aureus inhibition and biofilm resistance, while promoting osseointegration^19^. Both polydopamine and zwitterionic materials offer complementary benefits for implant surface modification. polydopamine ensures strong adhesion and biocompatibility, while zwitterions provide superior antifouling through hydration barrier formation. Combining these materials may synergistically enhance antibacterial properties without compromising mechanical strength. This study therefore aims to synthesize and optimize a polydopamine-based zwitterion nanoparticle coating on titanium and evaluate its physical and antibiofouling properties.

## Materials and methods

This interdisciplinary prospective in-vitro study was initiated following approval from the institutional ethical committee (Ref No. 18103). The effective sample size was calculated using G*Power (version 3.1.9.7) based on 0.05 significance, 0.8 power with confidence interval at 95%, accounting for a total 20 samples, divided into two groups: Group 1 consisted of titanium discs coated with polydopamine (PDA), while Group 2 included titanium discs coated with a combination of PDA and zwitterionic nanoparticles.

### Sample preparation

For the preparation of coatings, dopamine hydrochloride was procured from GenNext India, and titanium discs were obtained from SK Surgicals India. Distilled water required for the procedures was prepared using the Direct-Q^®^ and Direct-Q UV water purification system. Titanium disks (10 mm diameter, 2 mm thickness) were smoothed up to a surface roughness (Ra) under 40 nm. Once polished, samples were cleaned with isopropanol, ethanol, water, and acetone for 15 min each by sonication.

### Synthesis of polydopamine (PDA)–zwitter ionic (ZI) coating material & surface treatment procedure

Poly (MBAAm-co-SBMA) zwitter ionic nanoparticles were synthesised using the distillation-precipitation polymerisation technique, where N-(3-Sulfopropyl)-N-methacroyloxyethyl- N, N-dimethylammonium betaine (SBMA) was used as monomer and N, N΄- Methylenebisacrylamide (MBAA) was used as crosslinker as per the methodology described Bai et al.^20^, and Syed et al.^21^ Distillation-precipitation polymerization (DPP), a polymerization technique that was established by Feng et al., is the easiest to use^22^. It is a special technique that produces uniformly sized and shaped nanoparticles without the need of stabilizers or surfactants. Furthermore, as the refluxing solvent provides efficient mixing and an oxygen-free environment, this process can be scaled up. When compared to traditional polymerization methods like radical polymerization, group transfer polymerization, atom-transfer radical polymerization, and catalytic chain transfer polymerization, DPP offers more benefits, including shorter reaction times (usually 2–4 h), inexpensive starting materials, the absence of ligand and metal catalyst requirements, atmospheric conditions for the reaction, atom economy, and simple isolation techniques. The DPP mechanism is based on the sequence of radical initiation of the monomer or cross-linker, followed by chain addition and chain propagation, which precipitates the polymeric nanomaterial.

### Preparation of the coating material including zwitter ionic (ZI) and polydopamine (PDA)

**Fig. 1:**
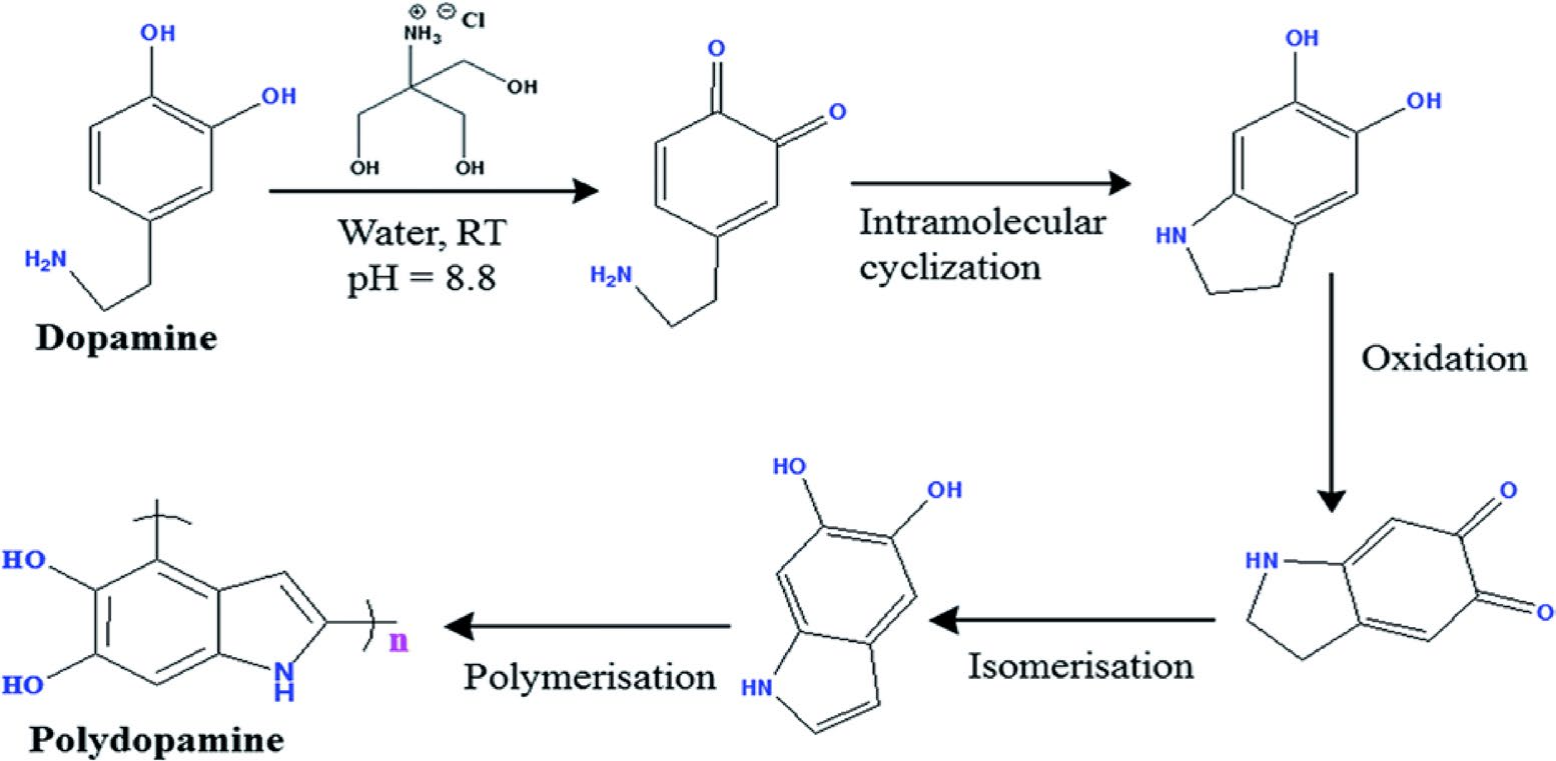
Polymerization of dopamine in aqueous media.

An initial step towards the synthesis of PDA-ZI coating material involved the preparation of hydroxymethyl aminomethane solution, also known as tris buffer in distilled water. The pH of the solution was brought to 8.8 with a 0.05 M tris solution in distilled water, which is a necessary condition for dopamine to undergo polymerization. For this step, a reagent vial was filled with 25 mL of the distilled water and the pH was adjusted to 8.8 by adding tris solution dropwise. A magnetic bead added. The reagent bottle was maintained at room temperature and subjected to mechanical agitation of 200−250 rpm. After each dropwise addition, the aqueous media’s pH was measured with a digital pH meter. Following the pH correction, Dopamine hydrochloride (25 mg) was weighed into it. When dopamine is dry, it ranges in color from white to yellow, which on polymerization turns black. The pyrocatechol stripe of dopamine is subjected to oxidation when it is consumed by dissolved oxygen found in an aqueous medium (pH 8.8). Pyrocatechol and benzoquinone undergo a disproportionation process that yields a semiquinone radical. A coupling reaction then occurs, resulting in the formation of polydopamine. Figure 1 provides the entire mechanism of polymerization.

After the polydopamine was formed, 5% (1.25 mg) zwitterion nanoparticles were weighed into the same reagent bottle To enable in-situ coating, titanium discs were added to the reaction mixture after roughly a minute. The reaction was kept under mechanical agitation for the next 24 h^11,23^. The samples were then dried in a vacuum oven for the next 24 h. The metal discs were checked and then prepared for additional characterization.

### Surface analysis

For the five samples from each of the PDA and Zwitter-PDA groups, FESEM and EDS (Scanning Electron Microscope with Energy Dispersive Analysis of X-rays), Zeta Potential, Particle Size Determination, and Atomic Force Microscopy were studied. The samples’ SEM and EDS results were acquired from the CIF Innovation Centre,. Additionally gathered and examined were the particle size, zeta potential, and Atomic Force Microscopy from the Central Research facility, NITK, Suratkal. The elemental mapping analysis was carried out to confirm the presence of all the elements.

###  Antimicrobial analysis: antimicrobial properties of the treated surfaces & test for antimicrobial efficacy

The Streptococcus mutans (MTCC 497) strains used in the study were obtained from Microbial Type Culture Collection and Gene Bank, Chandigarh, India). S.mutans was grown and was used to test the antimicrobial activity of two discs in each of the two groups (PDA & Zwitter ion + PDA). The organism was grown in 5 m Brain heart infusion (BHI, Difco, Detroit, MI, USA) broth at 37° C for 24 h in a CO_2_ incubator with 5% CO_2_. Every disc was inserted into a tissue culture plate well. The plate was incubated at 370 for 24 h after 1 milliliter of BHI broth containing 1.5* 10^8^ cfu/ml of Streptococcus mutans that had been cultured for 24 h was added. The disks were then twice cleaned in sterile PBS (PH–7.4). Washing of the discs before colony counting was done carefully, taking care so that the biofilm was not disrupted. Using a pipette, the broth was carefully aspirated from the tissue culture wells containing the disc, taking care not to touch the disc. Sufficient volume of sterile phosphate-buffered saline was added to the wells so that the discs were immersed. The plate was gently agitated for 1–2 min manually, after which the PBS was aspirated and discarded. This step was repeated, and then the discs were subjected to the colony counting method. For this, each disc was transferred to a sterile centrifuge tube, and 200 µl of PBS was added and vortexed for 2 min. Then logarithmic dilutions of the PBS was performed and plated onto BHI by the surface plating method. After vortexing the discs in 1 milliliter of PBS to separate the bacteria, the colonies were counted on BHI Agar at dilutions of 1:10,000, 1:100, and 1:1000^24^.

Each disc was inserted into a tissue culture plate well and 1mL of the broth culture adjusted to 0.5 Mac Farland standard (10^8^ colony forming units per mL) was added to each well containing the disc. The plate was incubated at 37° C for 24 h in the CO_2_ incubator (Nuaire, Plymouth, USA) with 5% CO_2_. Each disc was then rinsed twice with sterile phosphate-buffered saline (PBS pH 7.2). The disc was transferred to a sterile centrifuge tube and vortexed in 1 mL PBS to detach the adherent bacteria. Logarithmic dilutions (1:10, 1:100, 1:1000) of PBS was done and colony counting was performed by surface plating method on BHI agar (Ref: The plates were incubated at 37° C for 48 h in a CO_2_ incubator with 5% CO_2_. The number of colonies on each plate was counted and multiplied by the dilution factor to obtain the number of colony-forming units/mL. All the experiments were done in triplicate in a Biosafety level II Laboratory with ESCO Class II Biosafety cabinet, to ensure sterile conditions^11,25,26^.

###  Biological characterization of the surfaces

Human gingival fibroblasts (School of Life Science, Manipal) were used as a cell model to investigate the effects of surface and material variations on soft tissue response. Fibroblast was cultured on polished titanium discs at different time periods (3, 24, 48 h, 3, 4, 6, and 7 days) to determine the plating time. The growth curve derived from this data was used to assess the growth of fibroblasts between day 3 and day 6. Based on these results, the days showing an acceptable amount of growth were chosen to carry out the experiments. Appropriate volumes of Fibroblasts growth medium (DMEM - Dulbecco’s Modified Eagle’s Medium), EDTA in HBSS (0.53 mM), 0.04% trypsin in EDTA-HBSS, and growth medium and stop medium(10% fetal bovine medium) was prepared. Appropriate standard wells were selected and volume of the mediums was used accordingly^11^^[,25^.

The modified titanium discs were placed in a standard well tissue culture plate, and cells were seeded and growth medium was added into the well. The medium was removed by aspiration and EDTA was added carefully so as not to dislodge cell sheet. The cells were left in EDTA for up to 10 min. The cells were observed through the microscope. EDTA solution was removed and trypsin was added and incubated at 37 °C with 5% CO^2^ for 4 to 7 min. The side of the well was lightly tapped to encourage detachment. Trypsin was inactivated by washing the sides of the well with stop medium. Following this the wells were reseeded and incubated at 37 °C with 5% CO^2^^11,25^.

### Cell viability assay

The titanium discs were surface sterilized by exposing to UV in a class II biosafety cabinet hood (Esco Lifesciences, Singapore). The discs were then transferred to a 24-well cell culture plate. Gingival fibroblast cells were seeded at a density of 1 × 10^4^ on the surface of the titanium discs and allowed to adhere followed by the addition of DMEM containing 10% FBS for the indicated time in a sterile CO^2^ incubator. At the end of the indicated time points, the cells were lysed using RIPA cell lysis buffer. From the cell lysates, the protein concentration was estimated using a BCA protein assay kit (Thermo Fisher Scientific, USA) according to the manufacturer’s protocol. Briefly, reagents A and B were mixed in a 50: 1 ratio, and 200uL of this solution was mixed with 25uL of protein samples. This was incubated in a microcentrifuge tube for 30 min at 37^0^ C and color developed was read at 562 nm using a multimode reader (Thermo Fisher Scientific, USA). The cell viability of gingival fibroblast on the surface of the titanium disc was assessed by culturing the cells on their surface followed by performing protein estimation^11^. The experiments were performed in triplicates and repeated thrice independently. The data was represented as mean of independent experiments conducted.

### Corrosion studies

The corrosion nature of the both PDA and Zwitterionic coated titanium discs were evaluated using Electrochemical Impedance Spectroscopy (EIS) and Potentiodynamic Polarisation (Tafel) techniques in an artificial saliva medium. EIS analysis was conducted to obtain Nyquist plot, that provided a comparison of the charge transfer resistance in-order to assess the protective efficiency of the two coatings. Similarly, potentiodynamic polarisation were conducted to determine key electrochemical parameters such as corrosion current density i(corr) and corrosion potential E(corr), to analyse the corrosion tendencies between the two samples. All the analysis were carried out using a standard three electrode electrochemical cell, with coated titanium disc as the working electrode. The results of the analysis were used to asses and compare the corrosion resistance provided by the PDA and zwitterionic coating.

### Ethics approval

This study was performed in line with the principles of the Declaration of Helsinki and was approved by the Institutional Ethics Committee of Manipal College of Dental Sciences, Mangalore (ref. no.: 18103).

## Results

### Surface morphological analysis

**Fig. 2:**
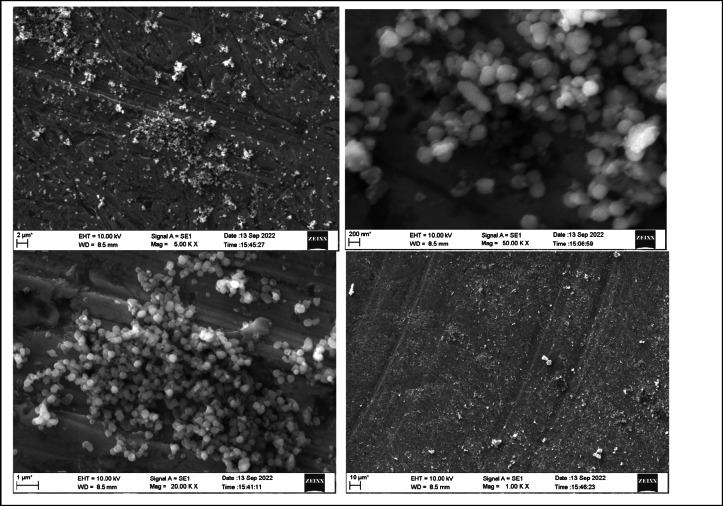
Field emission scanning electron microscope (FESEM) images of Polydopamine nanoparticle at 10 μm, 2 μm,1 μm and 200 nm.

**Fig. 3:**
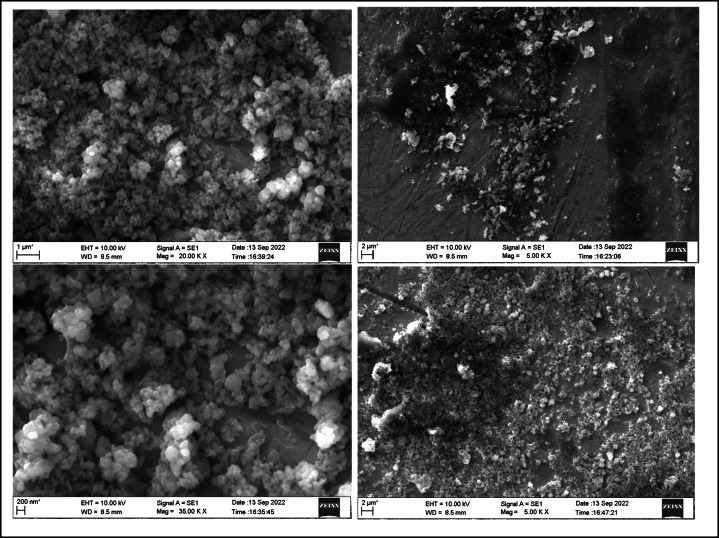
FESEM images of Zwitter nanoparticle on PDA at 2 µm,1 µm and 200 nm.

The morphology of the nanoparticles were visualized using a field emission scanning electron microscope (FESEM) and surface roughness parameters were studied using Atomic force microscopy. As shown in Figs. 2 and 3, uniform nanoparticle uptake was seen on the SEM images for both zwitter ion nanoparticles and polydopamine coating. morphology of the sample in comparison showed globular deposits which had a homogeneous distribution on the titanium surfaces.

**Fig. 4:**
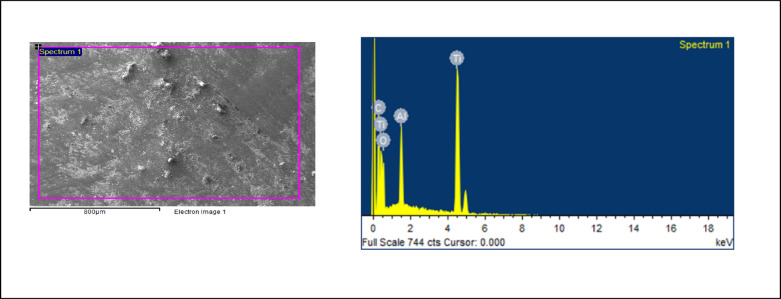
EDS images of Polydopamine nanoparticles.

**Fig. 5:**
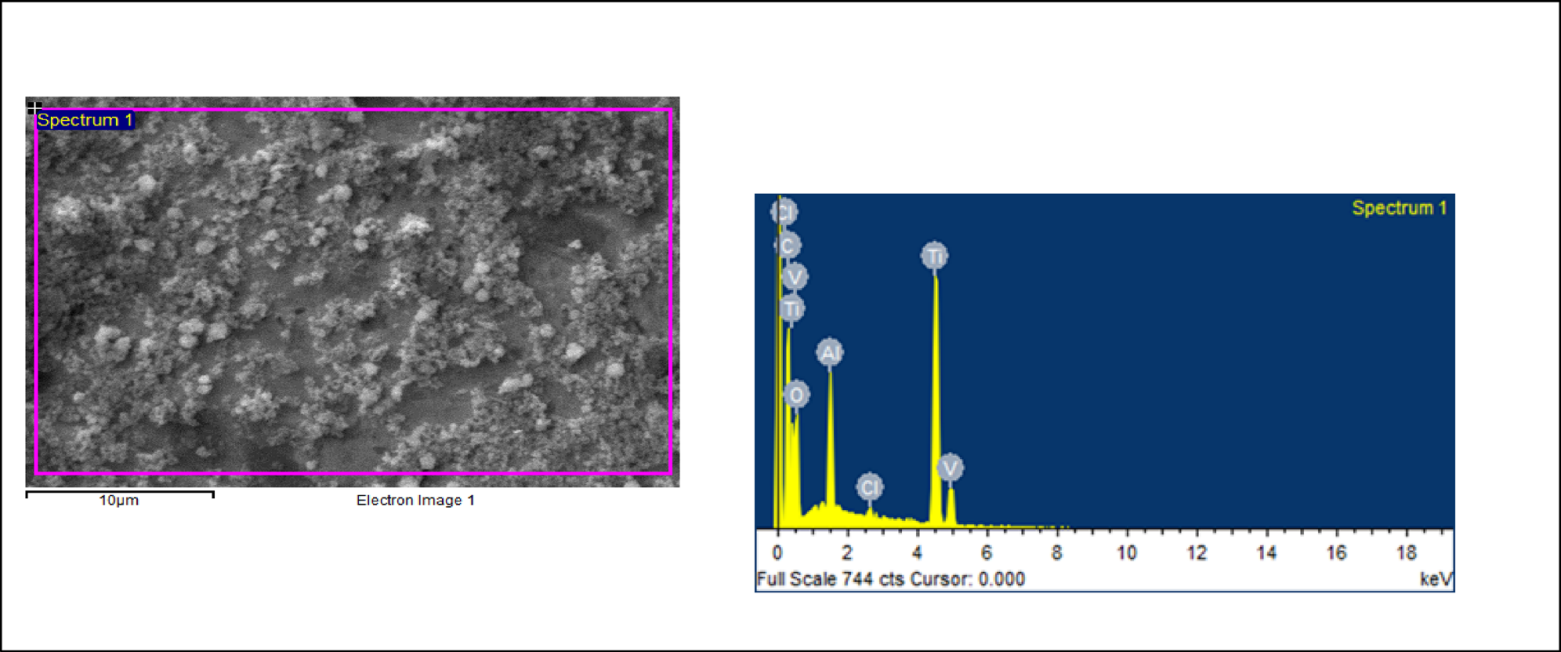
EDS images of Zwitter nanoparticles

Chemical Composition by Energy Dispersive Spectroscopy: Figs. 4 and 5; Tables 1 and 2, show the distribution of C, O, Al and Ti elements. The atomic composition of all surfaces was studied by means of EDS (Tables 1 and 2). For all the conditions, The oxygen and nitrogen signals remained the same, but an increase in the percentage of C ions was observed for the Zwitter ion samples. A similar level of Ti 2p signal was measured for both samples.

**Table 1:** PDA Elemental Composition.

Element	Weight %	Atomic %
C K	10.13	24.84
O K	14.12	26.02
Al K	5.32	5.81
Ti K	70.43	43.33

Peak possibly omitted : 2.605 keV, Processing option : All elements analyzed (Normalised)

**Table 2:** Zwitter Elemental Composition.

Element	Weight %	Atomic %
C K	15.01	31.84
O K	19.55	31.14
Al K	5.05	4.77

No peaks omitted, Processing option : All elements analyzed (Normalised)

**Fig. 6:**
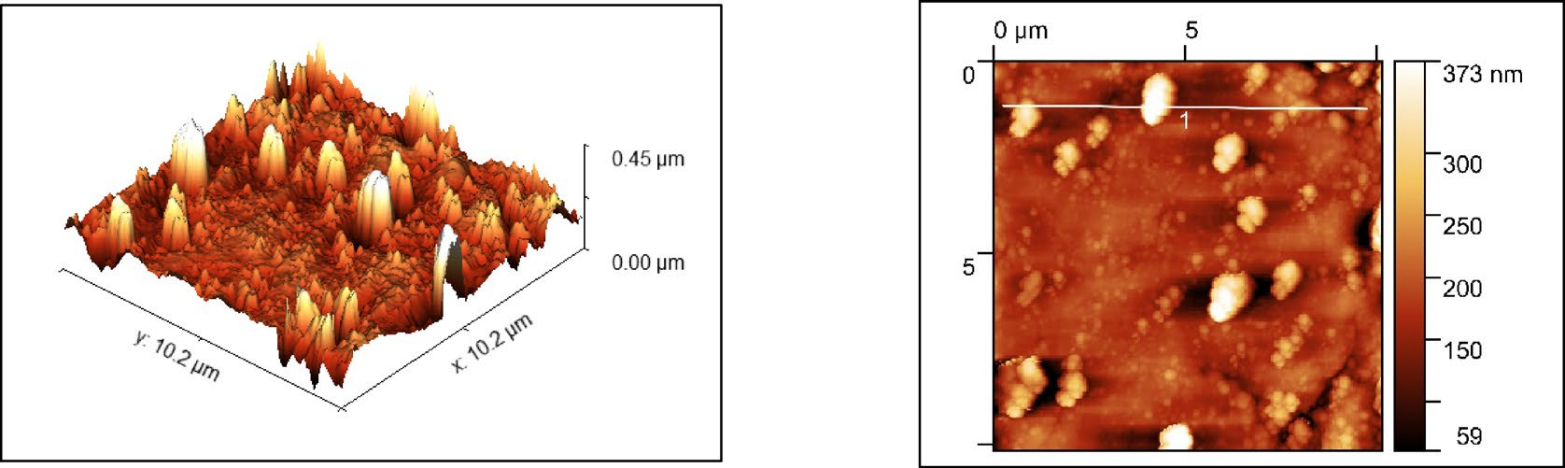
AFM Images of PDA.

**Fig. 7:**
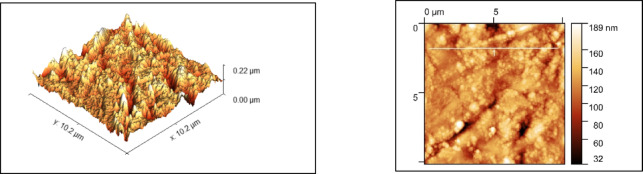
AFM Images of PDA+Zwitter samples.

The surface roughness values for PDA was around 373 nm and for PDA + Zwitter was around 189 nm (Figs. 6 and 7).

**Fig. 8:**
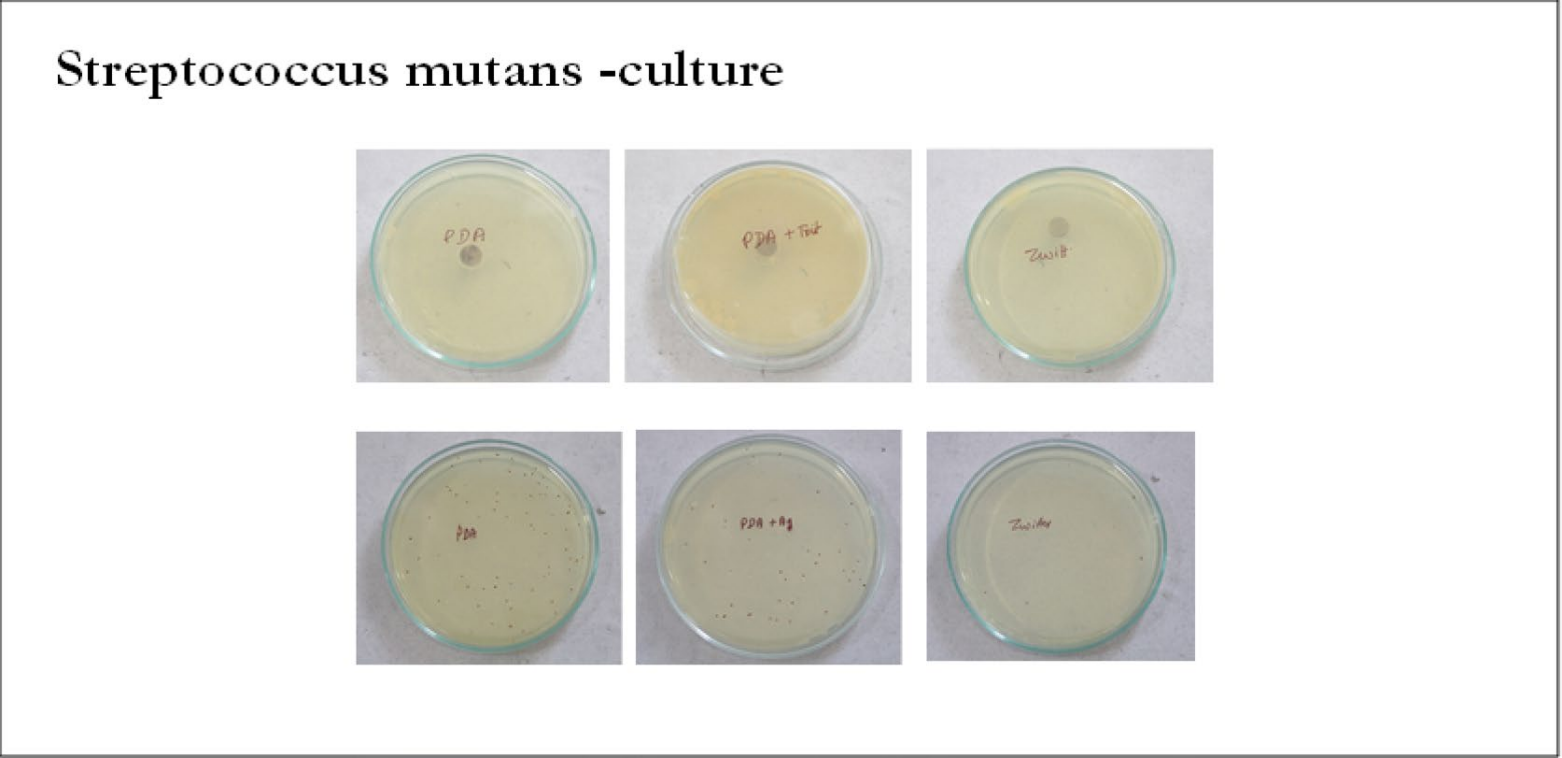
Shows the antibacterial activity of zwitter ion nanoparticles against the strain of *Streptococcus mutans* using the disk diffusion method.

**Table 3:** The Streptococcus mutans colony counts were as follows:

Nanoparticle disc	Colony count (cfu/mL)
Zwitter ion	1.25*10 ^4^ CFU/ml
PDA	1.4*10 ^5^CFU/ML
Difference	1.2*10^5^CFU/ML
t statistics, p value	-98.61, <0.001

Zwitter ion plate exhibited much lower average colony counts compared to PDA plate (Fig. 8) and this difference was statistically significant by Independent t test (*p* < 0.001).

### The cell viability

**Fig. 9:**
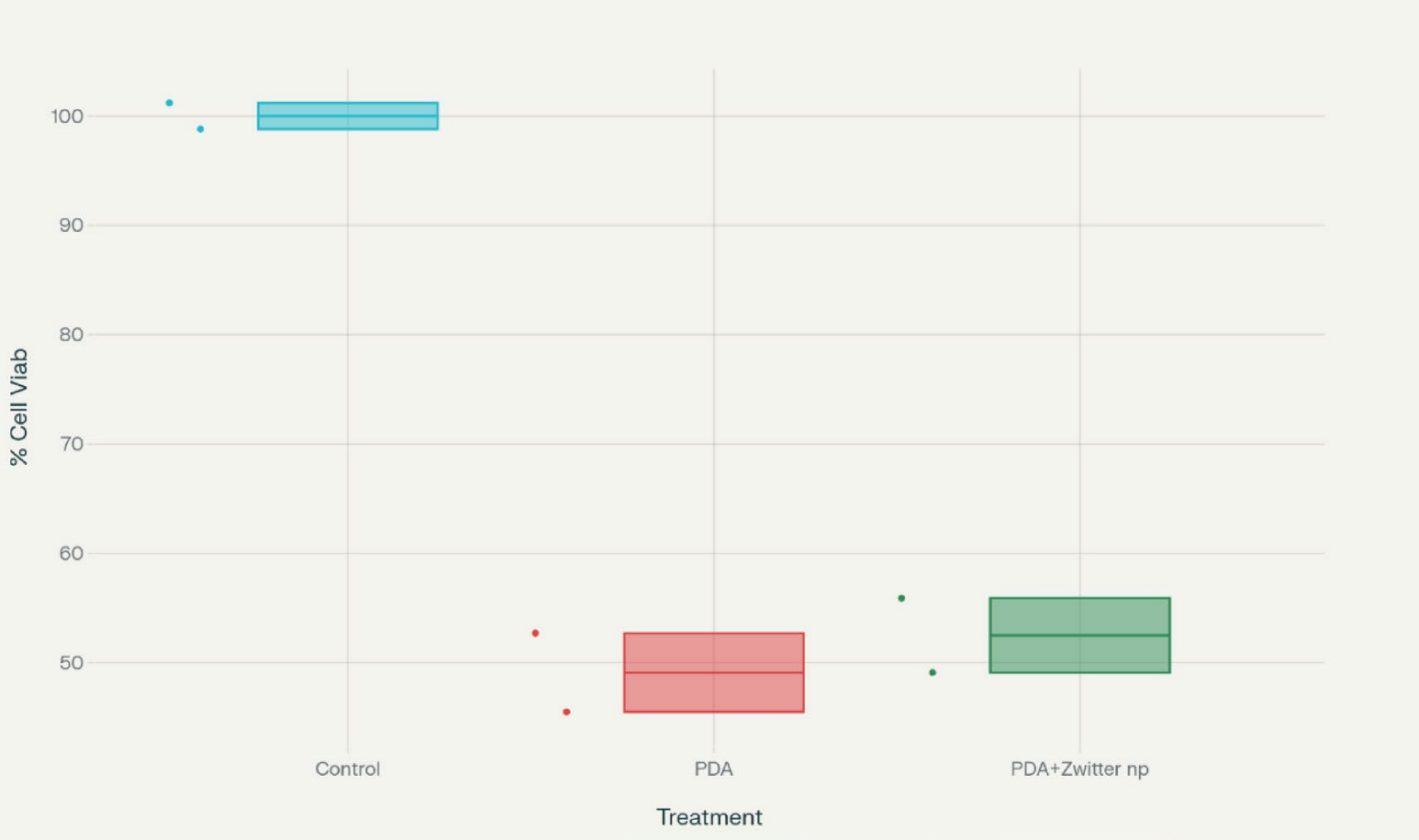
Box-Whisker Plot showing % of cell variability across three groups.

**Fig. 10:**
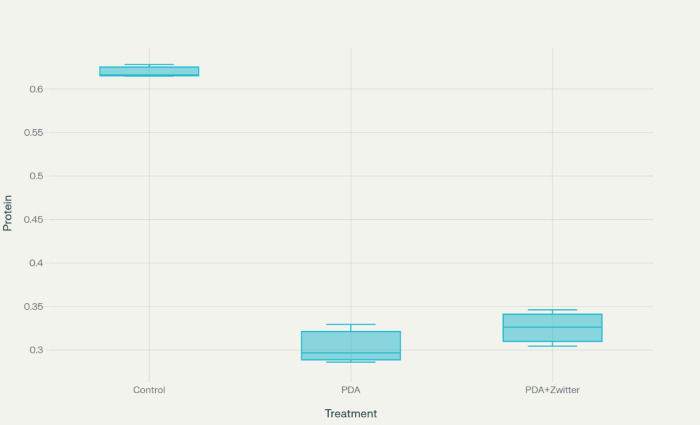
Box-Whisker Plot showing protein estimation across three groups.

The cell viability on the surface of PDA and PDA + Zwitter np were 49.1% and 52.5%, respectively. The viability or growth of the cells were also almost similar in PDA and PDA + Zwitter (poly (MBAAm-co-SBMA) np with PDA + Zwitter(poly (MBAAm-co-SBMA) np showing a slightly higher percentage of growth than plain PDA surface but not significant (Tables 4 and 5; Figs. 9 and 10).

**Table 4:** The % cell viability assessment between PDA & zwitterionic (poly (MBAAm-co-SBMA)) Nanoparticles.

Samples	% average	Std dev
Control	100.0	1.2
PDA	49.1	3.6
PDA + Zwitter np	52.5	3.4
Krushkal Wallis value, p value	4.57, 0.10	

**Table 5:** Protein estimation.

Samples	Protein reading		
Control	0.6162	0.6283	0.6148
PDA	0.3294	0.2861	0.2967
PDA + Zwitter np	0.3263	0.3462	0.3044
Kruskal-Wallis Value, P value	5.98, 0.05		

Both PDA and PDA + Zwitter ion nano particle treatments showed a decreased % cell viability compared to control group. But, this decrease wasn’t statistically significant (*p* = 0.1) by the Kruskal-Wallis test.

Even though PDA and PDA + Zwitter ion exhibited a lesser protein reading compared to control group, the difference between PDA and PDA + Zwitter ion is negligible and this difference is not statistically significant (*p* = 0.05) by the Kruskal-Wallis test. Between three readings also, there was no significant difference as tested by Friedman’s test for repeated measures. (*p* = 0.3)(Figs. 9 and 10).

### Corrosion studies

Corrosion behaviour of both PDA and Zwitterionic coated Titanium discs were studied through Electrochemical Impedance Spectroscopy (EIS) and Potentiodynamic polarisation taking artificial saliva as the medium, that have been discussed below.

### Electrochemical impedance spectroscopy (EIS)

**Fig. 11:**
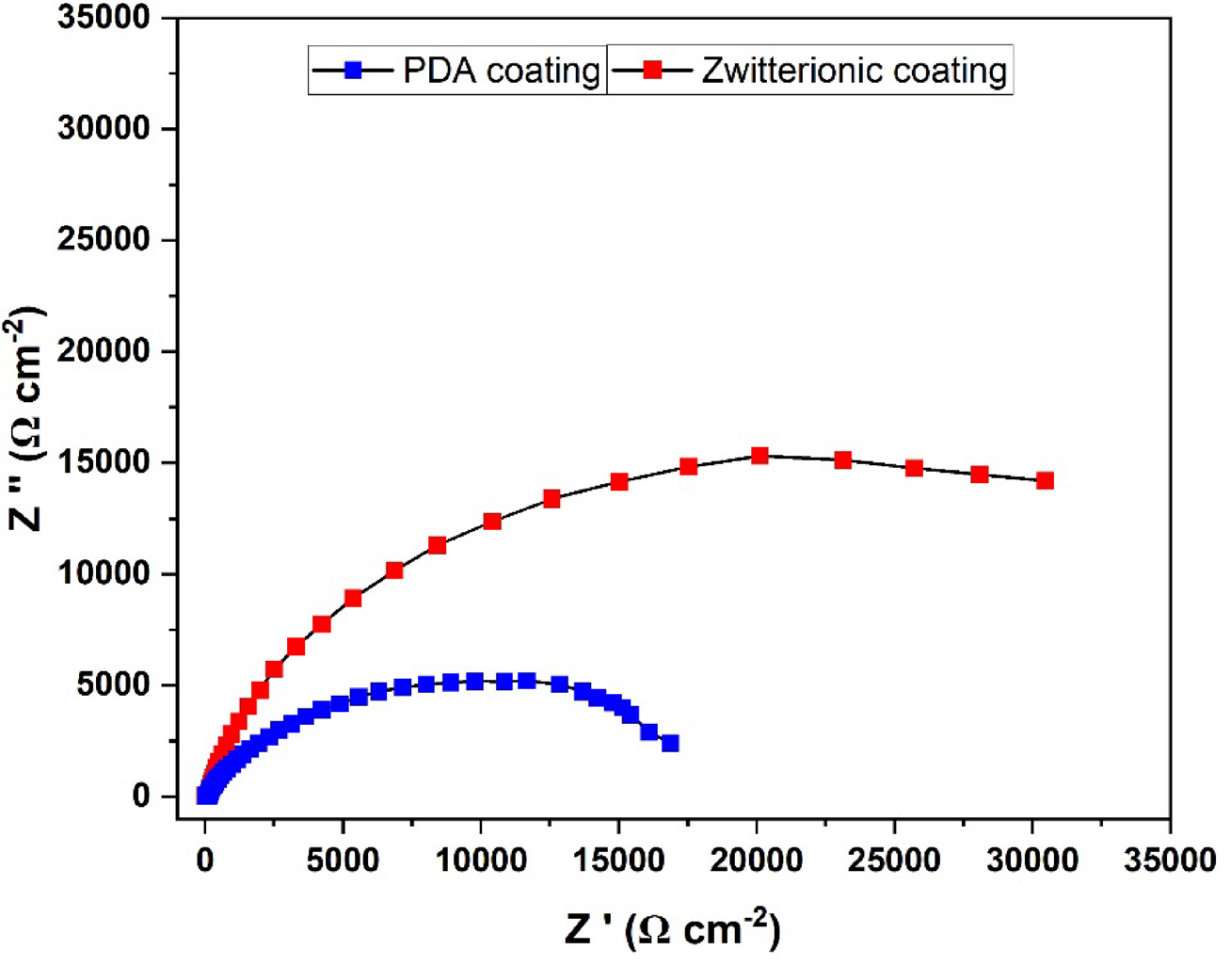
Nyquist plot Z″ vs. Z′ for PDA and Zwitterionic coated titanium discs.

The results of the EIS analysis of both PDA coated and zwitterionic coated titanium disc are presented the Fig. 11, which is also referred as the Nyquist plot. According to the obtained graph, the zwitterionic coating displays a larger charge transfer resistance semi-circle, indicating a significantly higher charge transfer resistance (R_ct_). This enhanced resistance directly translates to the higher corrosion resistance compared to the PDA coating^1,2^. The improved resistance to charge transfer is likely due to the formation of a strong hydration layer by the zwitterionic groups, that effectively delays corrosion initiation and progression^3^.

### Potentiodynamic polarisation analysis

**Fig. 12:**
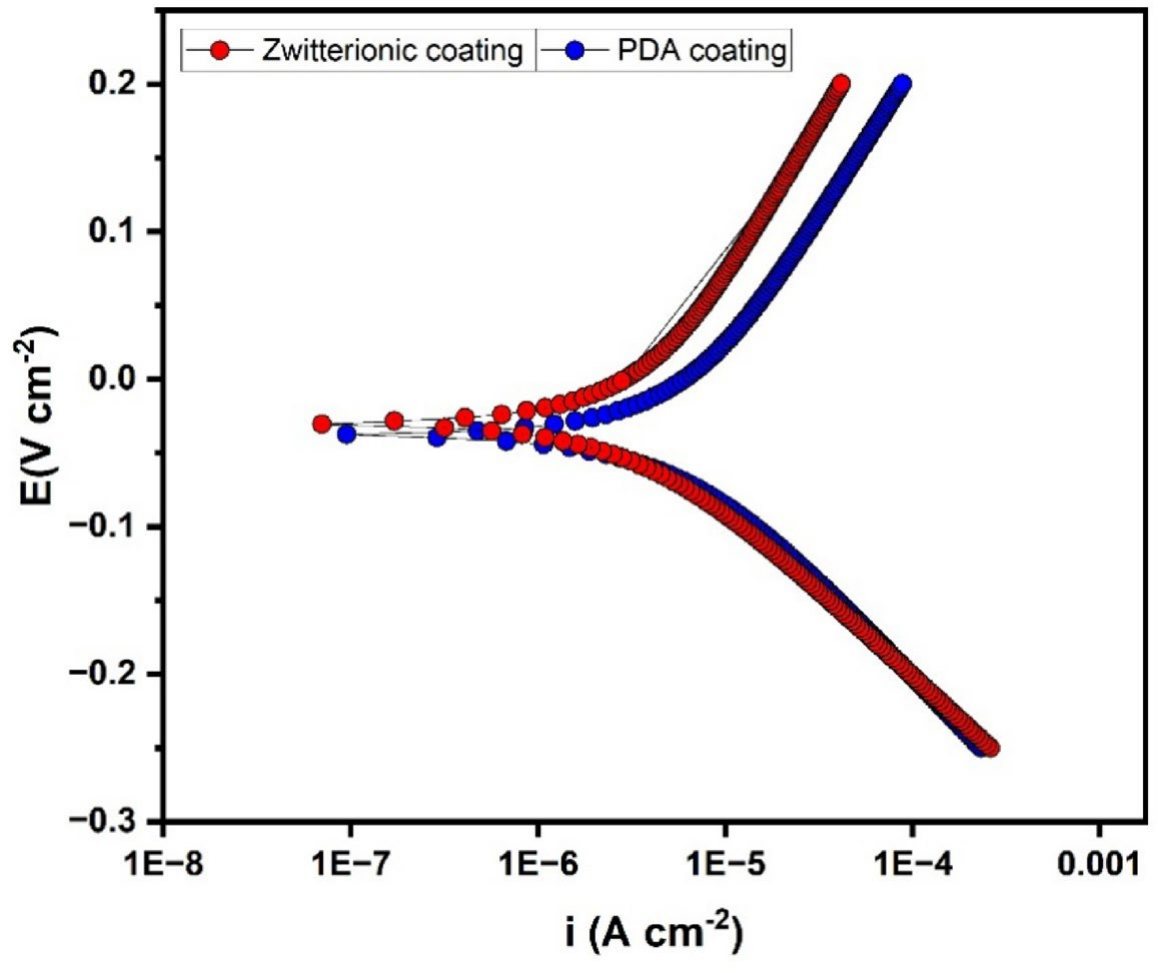
Tafel plot for the Zwitterionic coated and PDA coated titanium discs.

The results obtained from the Potentiodynamic polarisation (Tafel plot) are presented in Fig. 12. Using this plot, parameters like corrosion current density i(corr) and corrosion potential E(corr) were calculated and tabulated in (Table 6). The zwitterionic coated disc exhibited a lower corrosion current density, i(corr) of 3.55 × 10^− 6^ Acm^− 2^ compared to 5.92 × 10^− 6^ Acm^− 2^ for the PDA coated disc, stating that the zwitterionic coating corrodes slowly. Similarly, the zwitterionic coated disc also displayed a slightly noble or positive corrosion potential, E(corr) that the counterpart, confirming that the zwitterionic coated disc is less likely to initiate corrosion than the other^2^. Considering both the corrosion current density i(corr) and corrosion potential E(corr) values, it is clear that zwitterionic coated titanium disc exhibits enhanced corrosion resistance than the PDA coated disc^3^.

**Table 6:** Corrosion parameters obtained from the potentiodynamic polarisation analysis.

Parameters	PDA coated disc	Zwitterionic coated disc
Corrosion potential, E(corr)	−0.038 V	−0.03 V
corrosion current density, i(corr)	5.92 × 10^− 6^ Acm^− 2^	3.55 × 10^− 6^ Acm^− 2^
Anodic Tafel slope	0.203 V	0.215 V
Cathodic Tafel slop	−0.133 V	−0.118 V

From both the EIS and Potentiodynamic polarisation results, it is evident that the zwitterionic coated disc offers higher corrosion resistance and further signifying that the zwitterionic coating provides better protection than the PDA coating.

## Discussion

Biofilm control is a very pertinent issue in prevention and treatment of hospital related infections, especially in clinical dentistry and to counter this a lot of research has gone into the incorporation of various antimicrobial products ranging from adhesives to dental implants. Various types of antimicrobial strategies have been suggested to reduce or avoid the effect of biofilms^9^. Some of the strategies for the suppression of bacterial adherence ranges from prophylactic control of biofilm formation to the application of surface coatings on titanium. There are several coatings that has been has been experimented and antibacterial coatings has been a keen area of material research. Nanocoating’s are one such area of and the knowledge about this technology have changed the way the materials react with the oral tissues.

The primary objective of this research was to coat titanium, the common dental implant material with zwitter ion polydopamine nano particles. The process of distillation-precipitation polymerization (DPP) was as described by Feng et al.^8^, which is a relatively easier method of preparation of nanoparticles of comparatively regular size and shape. DPP method when compared to other classical polymerization processes such as atom-transfer radical-polymerization, group transfer polymerization, catalytic chain transfer polymerization, and radical polymerization, shows greater positives like cheaper material requirements, no requirement for catalyst and ligands, reduced reaction time and relatively easy isolation method. The mechanism of DPP follows the order of radical initiation of monomer or cross-linker and subsequent chain propagation by chain addition, which results in precipitation of polymeric nanomaterial^16^.

Recent literature have explored the use of zwitterionic nanoparticles as coatings on titanium implants, aiming to improve their antibacterial properties and resistance to biofilm formation. Zwitterionic materials, which contain both positive and negative charges, create a hydrated layer on surfaces, minimizing protein adhesion and bacterial attachment due to their superhydrophilic nature. This characteristic is particularly beneficial for implants, as it reduces the risk of infection without hindering osseointegration. Because zwitterion nanoparticles carry both positive and negative charges simultaneously, their surface chemistry confers antibacterial effects on microorganisms. It offers a very strong barrier against protein adsorption, which lowers bacterial attachment and, as a result, reduces the formation of biofilms^14^. This is observed in this study as a reduced colony count on the zwitter coatings as compared to the polydopamine coatings (Fig. 8). The zwitter nanocoatings showed 1.25*10 ^4^ Cfu/ml compared to 1.4*10 ^5^ Cfu/ml (Table 3). Our data shows a log reduction of approximately 1.05. As per literature, the standard for a treatment to be defined as “antibacterial active” is at least a 3-log reduction (which means a 99.9% kill rate). In our study, modified zwitterionic surface shows a statistically significant reduction in bacterial adhesion compared to the polydopamine control (about a 90% reduction in raw numbers). However, with only a 1-log reduction, it does not meet the standard to be classified as a strongly “antibacterial active” surface in this specific test. The weak result (1-log reduction instead of the desired 3-log) is a common challenge in surface modification.

Zwitterionic surfaces primarily work by creating a super-hydrophilic, electrostatically neutral surface that forms a strong hydration layer via hydrogen bonding^27^. This layer resists the initial adhesion of proteins and bacteria (anti-fouling), but it does not actively kill bacteria. Any flaw in the coating, insufficient density of zwitterionic groups, or suboptimal orientation can compromise this resistance, allowing some bacteria to adhere. Our results suggest the coating is partially effective but not perfect.

The efficacy is highly dependent on a uniform, dense, and stable coating. Inconsistent polymerisation, thickness, or degradation in the biological environment can create micro-sites where bacteria can attach, leading to the CFU counts that were observed. The result is from a single-time-point adhesion assay. Zwitterionic surfaces might perform worse under dynamic conditions (e.g., fluid flow) or over longer periods where an initial small number of adhered bacteria can proliferate and form a biofilm, eventually colonising the entire surface. Our study’s “weak” result highlights a fundamental trade-off in implant technology: biocompatibility versus potent antimicrobial activity. Our zwitterionic approach sits on the safer, more biocompatible end of the spectrum. It aims to create a “stealth” surface that the body and bacteria ignore. The 1-log reduction is a positive sign that the principle works, but it needs optimization for clinical use. Because of their hydrophilicity, zwitterionic materials have low fouling properties because hydration layers form on their surfaces. Electrochemistry is vital in bacterial adhesion since it is primarily caused by electrostatic attractive forces resulting from adhesions^15^. The other approaches like Silver, QACs, Antibiotics are on the potent, aggressive end. They achieve high log reductions by killing microbes, but this often comes at the cost of potential toxicity to human cells or the promotion of microbial resistance^28,29^.

Several recent studies have highlighted the potential of nanoparticle-based and zwitterionic coatings in enhancing the performance and biocompatibility of dental materials. Panchami et al. (2021) reported that zwitterionic nanoparticles synthesized via distillation–precipitation polymerization significantly improved membrane hydrophilicity, water flux (up to 249.4 L/m²/h), and protein rejection (92.1% for bovine serum albumin), underscoring their antifouling and separation efficacy in biomedical applications^14^. In a related context, Ahuja et al. (2024) demonstrated that polymeric and nanoparticle-coated orthodontic brackets reduced cytotoxic and genotoxic responses in oral mucosal cells compared to conventional stainless-steel brackets, despite no statistically significant difference in toxicity levels, indicating improved biocompatibility^11^. Further, Rao et al. (2024) found that orthodontic brackets coated with a combination of TiO₂ nanotubes and methacryloyloxyethylphosphorylcholine exhibited superior antibacterial properties and the lowest surface roughness, effectively reducing *Streptococcus mutans* adhesion and potential for white spot lesion formation. Collectively, these findings support the integration of zwitterionic and nanoparticle-based coatings for enhancing surface functionality, biocompatibility, and antibacterial efficacy of dental biomaterials^25^.

In this study, a coating of polydopamine and its zwitter ion form on titanium has shown promising antibiofouling results, effectively deterring bacteria and supporting biocompatibility with human cells​^30^. cytotoxicity of the coated surfaces were studied with Humam gingival fibroblast adhesion and proliferation assays (Tables 3 and 4). The results did not reveal reduction in viable cells for up to 6 days of incubation. According to the International Organization for Standardization (ISO 10993-6:2007), reductions in cell viability of less than 20% are not considered cytotoxic^31,32^. Once established that none of the coatings were cytotoxic, the study focused on the cell viability using human gingival fibroblasts cell line. In this study, the effect of zwitternano coatings and polydopamine coatings were studied for bacterial adhesion and their ability to prevent or reduce biofilm formation which is similar to the findings of Wen.C et al. By incorporating these zwitterionic coatings through photopolymerization and other layer-by-layer methods, titanium implants can sustain prolonged antibacterial activity^33,34^. These findings can lead to a change in mindset, where zwitterionic nanoparticles can be utilised for their bioactive properties to support both patient safety and invivo implant longevity. This points toward future applications of biomimetic implants which can resist microbial growth all while integrating effectively with bone tissue. As the hospital settings are a multispecies environment, future in vitro studies should focus on the effect of polymicrobial models on bacterial adhesion to bioactive materials over longer periods of time.

## Limitations and future scope

This study was limited by its in vitro design, which may not fully replicate the complex biological environment of the oral cavity or other implantation sites. Long-term durability of the coatings and their behavior under mechanical loading or dynamic fluid conditions remain untested. Additionally, only a single bacterial species (*Streptococcus mutans*) was evaluated, which does not reflect the polymicrobial nature of biofilms associated with clinical infections. Long term research should focus on in vivo validation, extended antimicrobial spectrum testing, and assessment of mechanical stability and osseointegration potential to support clinical translation of the PDA-zwitterionic(poly (MBAAm-co-SBMA) nanoparticle coatings. Future studies could explore the integration of the anti-adhesive properties of zwitterionic coatings with low doses of non-toxic antimicrobial agents to develop a dual strategy.of resistance and elimination. Additionally, systematic optimization of coating parameters—including monomer concentration, reaction time, and degree of cross-linking—may help achieve a denser and more stable hydration layer.

## Conclusion

Zwitter ion nanoparticles (poly (MBAAm-co-SBMA) prepared by the distillation-precipitation polymerization method can be used as the antibacterial coating of choice in healthcare applications. These coatings show good surface characteristics compared to other nanocoatings and also reduce bacterial adhesion considerably. The antibacterial activity of zwitter ion nanoparticles against the strain of *Streptococcus mutans* using the disk diffusion method showed that poly (MBAAm-co-SBMA) Zwitter ion had better antibacterial properties (*S.mutans* colony count- 1.25*10 4 CFU/ml) compared to polydopamine PDA which showed a colony count of 1.4*10 5 CFU/ML. In summary, our results are not a failure but a realistic data point in the challenging field of antimicrobial surfaces. They correctly identify that a simple zwitterionic modification, while beneficial, is insufficient as a standalone solution for a highly “antibacterial active” implant and must be improved or combined with other strategies to meet the rigorous 3-log reduction standard. These nanoparticle coatings can be used to design materials that will resist biofilm formations and reduce infective processes thereby leading to better clinical outcomes.

## Data Availability

The datasets used and/or analysed during the current study available from the corresponding author on reasonable request.
